# A biomechanical mathematical model for the collagen bundle distribution-dependent contraction and subsequent retraction of healing dermal wounds

**DOI:** 10.1007/s10237-016-0821-2

**Published:** 2016-08-31

**Authors:** Daniël C. Koppenol, Fred J. Vermolen, Frank B. Niessen, Paul P. M. van Zuijlen, Kees Vuik

**Affiliations:** 1 Delft Institute of Applied Mathematics, Delft University of Technology, Delft, The Netherlands; 2 Department of Plastic, Reconstructive and Hand Surgery, MOVE Research Institute, VU University Medical Centre, Amsterdam, The Netherlands; 3 Burn Centre, Red Cross Hospital, Beverwijk, The Netherlands; 4 Department of Plastic, Reconstructive and Hand Surgery, Red Cross Hospital, Beverwijk, The Netherlands

**Keywords:** Dermal wound healing, Wound contraction, Collagen bundle-reinforced anisotropic soft tissue, Modeling, Biomechanics, 35L65, 35M10, 65C20, 68U20, 74L15, 92C10, 92C17

## Abstract

A continuum hypothesis-based, biomechanical model is presented for the simulation of the collagen bundle distribution-dependent contraction and subsequent retraction of healing dermal wounds that cover a large surface area. Since wound contraction mainly takes place in the dermal layer of the skin, solely a portion of this layer is included explicitly into the model. This portion of dermal layer is modeled as a heterogeneous, orthotropic continuous solid with bulk mechanical properties that are locally dependent on both the local concentration and the local geometrical arrangement of the collagen bundles. With respect to the dynamic regulation of the geometrical arrangement of the collagen bundles, it is assumed that a portion of the collagen molecules are deposited and reoriented in the direction of movement of (myo)fibroblasts. The remainder of the newly secreted collagen molecules are deposited by ratio in the direction of the present collagen bundles. Simulation results show that the distribution of the collagen bundles influences the evolution over time of both the shape of the wounded area and the degree of overall contraction of the wounded area. Interestingly, these effects are solely a consequence of alterations in the initial overall distribution of the collagen bundles, and not a consequence of alterations in the evolution over time of the different cell densities and concentrations of the modeled constituents. In accordance with experimental observations, simulation results show furthermore that ultimately the majority of the collagen molecules ends up permanently oriented toward the center of the wound and in the plane that runs parallel to the surface of the skin.

## Introduction

The overall healing of deep dermal wounds that cover a large surface area is an extremely complex process (Enoch and Leaper 2007; O’Toole and Mellerio 2010). Looking more closely at the healing process, the process can be split up into an interdependent series of relatively simpler subprocesses. One of these subprocesses is wound contraction. Wound contraction is a biomechanical process that causes the circumferential inward movement of surrounding uninjured tissue toward the wounded area (Baum and Arpey 2005; Monaco and Lawrence 2003). Due to this inward movement of the uninjured tissue, the exposed surface area of the wound can be decreased substantially and relatively fast without the production of new tissues. For instance, due to wound contraction, typical full-thickness wounds in humans may undergo a reduction in wound surface area of up to 40 % over a period of several weeks, while rapid wound contraction in rats may even be responsible for up to 90 % of the closure of the wounded area (Li et al. 2007; McGrath and Simon 1983).

Given the possible extent of wound contraction and the severity of related complications such as the development of permanent shortenings of scar tissue, a lot of resources have been allocated to the research on wound contraction over the last few decades. This research has resulted in the production of much knowledge about the biomechanical mechanisms underlying wound contraction. However, there is still much that remains understood incompletely about both the mechanisms underlying wound contraction and the etiology of the complications that may develop as a result of wound contraction. Given that fully adequate treatment plans for the prevention of, for instance, the development of permanent shortenings of scar tissue do not exist at the moment and a better understanding of the mechanisms underlying wound contraction most probably would aid in the development of better treatment plans, this is an unsatisfactory situation.

In order to help with gaining more insight into the mechanisms underlying wound contraction, Tranquillo and Murray (1992) formulated the first mathematical framework for the modeling of the mechanical component of the wound healing response. This framework is based on the continuum hypothesis and the conservation of both mass and linear momentum. In the resulting model, the dermal tissues are modeled as homogeneous, isotropic, linear viscoelastic solids and the fibroblasts that are present in these tissues produce an isotropic stress that works on these tissues. Interestingly, Tranquillo and Murray were able to replicate with this model the experimental data on wound contraction in rats that were collected by McGrath and Simon (1983). Subsequently, the general framework has been extended and has been adapted in several more recent modeling studies in order to investigate the impact of the addition and the adaptation of various different components of the wound healing response (Friedman et al. 2012; Javierre et al. 2009; Koppenol et al. 2016; Murphy et al. 2012; Olsen et al. 1998a, 1995; Tracqui et al. 1995; Ramtani 2004; Ramtani et al. 2002; Valero et al. 2013, 2014; Vermolen and Javierre 2012).

Recently, continuum hypothesis-based models have been formulated that are based on a morphoelastic framework (Bowden et al. 2016; Murphy et al. 2011). With these models, it is possible to simulate simultaneously both the contraction and the growth of involved dermal tissues during the execution of the overall healing process. This combination makes it possible to simulate the permanent deformation of these tissues and the development of residual stresses within these tissues.

Although the mathematical models in the above cited studies contain functional descriptions of several different components of the wound healing response, they all lack a description of the dynamic regulation of the geometrical arrangement of the constituents of the extracellular matrix (ECM). Given that the behavior of dermal tissues in response to mechanical forces such as wound contraction is influenced strongly by the geometrical arrangement of the constituents of the ECM (Wilkes et al. 1973), we think it is a limitation of these models that they lack such a description.

The ECM is the non-cellular component of dermal tissues and is composed of two classes of macromolecules: proteoglycans and fibrous proteins (Jarvelainen et al. 2009; Schaefer and Schaefer 2010). Taken together these macromolecules form a relaxed network of protein fibers embedded in a hydrated gel. The most abundant type of fibrous protein is collagen. These proteins provide most of the tensile strength to the tissues they are embedded in (Rozario and DeSimone 2010). The majority of the collagen molecules is produced by fibroblasts and is organized into interconnected sheets and bundles by these same fibroblasts (Monaco and Lawrence 2003). Due to this organization of the collagen molecules into interconnected sheets and bundles, the geometrical arrangement of the collagen bundles, in particular, has a huge impact on the response of dermal tissues to mechanical forces (Jor et al. 2011).

Barocas and Tranquillo (1997) formulated the first mathematical framework that includes a description of the dynamic regulation of the geometrical arrangement of collagen bundles in tissues. This framework is also based on the continuum hypothesis and the conservation of both mass and linear momentum. The ECM is modeled as a heterogeneous, anisotropic, biphasic medium consisting of a fibrillar network and an interstitial solution. The two phases display intraphase viscoelasticity and interphase frictional drag due to the relative motion of the two phases. The biphasic medium both orients and is oriented by fibroblasts. Toward a validation of the resulting model, Barocas and Tranquillo demonstrated qualitative agreement between model predictions on the one hand and various outcomes of experiments performed on tissue-equivalent systems on the other hand.

Subsequently, Olsen et al. (1998b, (1999) also formulated a continuum hypothesis-based general framework that includes a description of the dynamic regulation of the geometrical arrangement of either one type or two types of bundle. In this framework, the ECM is modeled as a heterogeneous, anisotropic, viscoelastic medium in which the bundle type(s) both orient(s) and is (are) oriented by fibroblasts.

Dallon et al. (1999, (2000, (2001) and McDougall et al. (2006) also developed a theoretical framework in order to study the dynamic regulation of the arrangement of collagen bundles. However, they took a different approach: cells are modeled as discrete entities, while the ECM is modeled as a continuum. Here too, the direction of movement of the cells is influenced by the local orientation of the collagen bundles, while the reorientation of the bundles is in turn dependent of the polarity of the cells in the vicinity. A couple of years ago, this hybrid framework has been extended by Cumming et al. (2010). More recently, we have extended the model developed by Cumming et al. on our part by adding a mechanical component to this model (Boon et al. 2016).

Although the frameworks and the associated models mentioned in the last two paragraphs are very elegant, they either do not contain a mechanical component at all or they lack an incorporation of the effect of the geometrical arrangement of the collagen bundles on the bulk mechanical behavior of the involved dermal tissues. Hence, these models are not able to simulate the direct influence of the composition and the topology of the constituents of the ECM on the behavior of dermal tissues in response to mechanical forces such as wound contraction.

Recently, two models have been developed that do incorporate the effect of the geometrical arrangement of the collagen bundles on the bulk mechanical behavior of the tissues these bundles are embedded in (Valero et al. 2015; Yang et al. 2013). However, in our opinion, each of these two models has a serious limitation. Due to the fact that the model developed by Yang et al. uses a hybrid framework similar to that developed by Dallon et al. (1999), the domain of computation, and hence the created wounds, has to be small so that the computation times and the computer memory requirements remain acceptable. Therefore, they used a domain of computation of solely $4 \, \text {mm}^{2}$ and created circular wounds with a radius of $400\ \upmu \text {m}$. However, if dermal wounds cover such a small surface area, then wound contraction actually does not play a very substantial role during the healing process. Because of the fact that the model developed by Valero et al. is a continuum hypothesis-based model, it is possible to simulate the healing of deep dermal wounds that cover a large surface area with this model. However, this latter model lacks a dynamic regulation of the geometrical arrangement of the collagen bundles. Given that, for instance, the proportion of the collagen bundles that runs parallel to the surface of the skin in general increases considerably due to the execution of the wound healing processes (Welch et al. 1990), we consider it a serious limitation that the dynamic regulation of the geometrical arrangement of the collagen bundles is not included in the model developed by Valero et al.

Because of the fact that we wish to simulate the contraction of deep dermal wounds that cover a large surface area where the contraction process is influenced by the geometrical arrangement of the collagen bundles, and given the aforementioned limitations associated with the models developed by Yang et al. and Valero et al., we developed a new, fully continuum hypothesis-based model for the simulation of the contraction and the subsequent retraction of healing dermal wounds. In this model, the bulk mechanical behavior of the involved dermal tissues is dependent on the geometrical arrangement of the collagen bundles. For this end, we use a tensorial approach similar to the one proposed by Barocas and Tranquillo (1997) and Cumming et al. (2010) to represent the collagen bundles, and we let the bulk mechanical properties of the tissues such as the Young’s moduli and Poisson ratios depend locally on both the local concentration and the local geometrical arrangement of these bundles. Furthermore, we incorporate into this new model the dynamic change of the geometrical arrangement of the collagen bundles similar to how this process is incorporated in the model developed by Olsen et al. (1998b). A detailed description of the full model is presented next in Sect. 2. In Sect. 3, we give a short overview of the applied numerical algorithm for obtaining simulation results. The simulation results are presented in Sect. 4. Finally, the model and the simulation results are discussed in Sect. 5.

## Development of the mathematical model

In order to simulate the contraction and subsequent retraction of healing dermal wounds, we include into the model some of the subprocesses that take place during the proliferative and remodeling phase of the wound healing cascade (Enoch and Leaper 2007). With respect to the subprocesses that are executed during the proliferative phase, we select the following subprocesses: wound contraction and fibroplasia. (Fibroplasia encompasses the subprocesses that cause the restoration of the presence of fibroblasts and the restoration of a collagen-rich ECM in the injured area.) Since wound contraction mainly takes place in the dermal layer of the skin, solely a portion of this layer is included explicitly into the model.

The dermis is modeled as a heterogeneous, anisotropic continuous solid with bulk mechanical properties that are locally dependent on both the local concentration and the local geometrical arrangement of the collagen bundles. Due to the fact that the collagen bundles are represented by means of a symmetric tensor, the dermal layer has material properties that differ locally along three mutually orthogonal twofold axes of rotational symmetry. (The symmetry axes coincide with the lines that pass through the individual material points of the dermal layer and run parallel to the individual eigenvectors of the tensor that represents the collagen bundles. See Sect. 2.3 for further details.) Therefore, the dermal layer is actually modeled as an orthotropic material (Lai et al. 1999). With respect to the mechanical component of the model, the displacement of the dermal layer (${\mathbf {u}}$) is chosen as the primary model variable. Furthermore, we assume that it is appropriate to apply the infinitesimal strain theory in this study. Hence, we take

1 $$\begin{aligned} {\mathbf {e}} \approx {\mathbf {\epsilon }} = \frac{1}{2}\left[ \nabla {\mathbf {u}} + (\nabla {\mathbf {u}})^{\text {T}}\right] , \end{aligned}$$

where ${\mathbf {e}}$ is the Eulerian strain tensor and ${\mathbf {\epsilon }}$ is the infinitesimal strain tensor. Finally, we select the following four constituents of the dermal layer as primary model variables: fibroblasts (*N*), myofibroblasts (*M*), collagen bundles ($\Omega ^{\rho }$), and a generic signaling molecule (*c*).

The general continuum hypothesis-based mathematical framework of Tranquillo and Murray (1992) is used as basis for the model. This framework consists of the following general set of conservation equations in local form:

2a $$\begin{aligned} \frac{\partial z_{i}}{\partial t} + \nabla \cdot \left[ z_{i}{\mathbf {v}}\right]&= -\nabla \cdot \mathbf {J}_{i} + R_{i}, \end{aligned}$$

2b $$\begin{aligned} -\nabla \cdot \mathbf {\sigma }&= \mathbf {f}. \end{aligned}$$

Equation (2a) is the mass conservation equation for the cell density/concentration of constituent *i* of the dermal layer and Eq. (2b) is the reduced conservation equation for the linear momentum of the dermal layer. It is assumed that the inertial forces that work on the dermal layer are negligible, and therefore, the conservation equation for the linear momentum of the dermal layer reduces to the above force balance equation. Within the above equations, $z_{i}$ represents the cell density/concentration of constituent *i*, ${\mathbf {v}}$ represents the displacement velocity of the dermal layer, $\mathbf {J}_{i}$ represents the flux associated with constituent *i* per unit area, $R_{i}$ represents the (bio)chemical kinetics associated with constituent *i*, $\mathbf {\sigma }$ represents the Cauchy stress tensor associated with the dermal layer, and $\mathbf {f}$ represents the total body force working on the dermal layer. Given the chosen primary model variables, we have $i \in \{N,M,c,\Omega ^{\rho }_{jk}\}$ with $j,k \in \{1,2,3\}$. In the remainder of this text, we replace $z_{N}$ by *N*, $z_{M}$ by *M*, $z_{c}$ by *c*, and $z_{\Omega ^{\rho }_{jk}}$ by $\Omega ^{\rho }_{jk}$.

### The cell populations

The functional forms for the movement of the (myo)fibroblasts and the functional forms for the biochemical kinetics associated with these cells are identical to functional forms used previously (Koppenol et al. 2016). For completeness we present these functional forms here as well. More details about the functional forms can be found in the cited article. The functional forms for the cell fluxes are

3 $$\begin{aligned} \mathbf {J}_{N}&= -D_{F}F\nabla N + \chi _{F}N\nabla c, \end{aligned}$$

4 $$\begin{aligned} \mathbf {J}_{M}&= -D_{F}F\nabla M + \chi _{F}M\nabla c, \end{aligned}$$

where

5 $$\begin{aligned} F = N + M. \end{aligned}$$

Parameter $D_{F}$ is the cell density-dependent random motility coefficient of the (myo)fibroblast, and $\chi _{F}$ is the chemotactic coefficient. The functional forms for the biochemical kinetics associated with the (myo)fibroblasts are

6 $$\begin{aligned}&R_{N} = r_{F}\left[ 1 + \frac{r_{F}^{\max }c}{a_{c}^{I} + c}\right] [1 - \kappa _{F}F]N^{1 + q} - k_{F}cN - \delta _{N}N, \end{aligned}$$

7 $$\begin{aligned}&R_{M} = r_{F}\left[ \frac{\left[ 1 + r_{F}^{\max }\right] c}{a_{c}^{I} + c}\right] [1 - \kappa _{F}F]M^{1 + q} + k_{F}cN - \delta _{M}M, \end{aligned}$$

where $r_{F}$ is the cell division rate, $r_{F}^{\max }$ is the maximum factor with which the cell division rate can be enhanced due to the presence of the signaling molecule, $a_{c}^{I}$ is the concentration of the signaling molecule that causes the half-maximum enhancement of the cell division rate, $\kappa _{F}F$ represents the reduction in the cell division rate due to crowding, *q* is a fixed constant, $k_{F}$ is the signaling molecule-dependent cell differentiation rate of fibroblasts into myofibroblasts, $\delta _{N}$ is the apoptosis rate of fibroblasts, and $\delta _{M}$ is the apoptosis rate of myofibroblasts.

### The generic signaling molecule

The functional form for the dispersion of the generic signaling molecule and the functional forms for the release, the consumption, and the removal of the generic signaling molecule are also identical to functional forms used previously (Koppenol et al. 2016):

8 $$\begin{aligned} \mathbf {J}_{c}&= -D_{c}\nabla c, \end{aligned}$$

9 $$\begin{aligned} R_{c}&= k_{c}\left[ \frac{c}{a_{c}^{II} + c}\right] \left[ N + \eta M\right] - \delta _{c}\left[ \frac{\text {tr}\left( \Omega ^{\rho }\right) Fc}{1 + a_{c}^{III}c}\right] . \end{aligned}$$

The parameter $D_{c}$ represents the random diffusion coefficient of the generic signaling molecule, $k_{c}$ represents the maximum net secretion rate of the signaling molecule, $\eta$ is the ratio of myofibroblasts to fibroblasts in the maximum net secretion rate of the signaling molecule, $a_{c}^{II}$ is the concentration of the signaling molecule that causes the half-maximum net secretion rate of the signaling molecule, $\delta _{c}$ is the proteolytic breakdown rate of the signaling molecules, $1/(1 + a_{c}^{III}c)$ represents the inhibition of the removal of signaling molecules and collagen molecules (See Sect. 2.3) due to the presence of the signaling molecule, and $\text {tr}\left( \Omega ^{\rho }\right)$ represents the concentration of the collagen molecules (See Sect. 2.3).

### The collagen bundles

As has been mentioned in the introduction, we use a tensorial approach similar to the one proposed by Barocas and Tranquillo (1997) and Cumming et al. (2010) to represent the collagen bundles. The orientation of the collagen bundles and the concentration of the collagen molecules at location $\mathbf {x}$ and time *t* within the dermal layer are represented together by the symmetric tensor

10 $$\begin{aligned} \Omega ^{\rho }(\mathbf {x},t)= & {} \int _{0}^{\pi /2}\int _{0}^{\pi }\nonumber \\&\left[ \mathbf {p}(\theta ,\varphi )\left( \mathbf {p}(\theta ,\varphi )\right) ^{\text {T}}\rho (\mathbf {x},\theta ,\varphi ,t)\right] {\text {d}}\theta {\text {d}}\varphi ,\nonumber \\ \end{aligned}$$

with $(\mathbf {p}(\theta ,\varphi ))^{\text {T}} = [\sin (\varphi )\cos (\theta ),\sin (\varphi )\sin (\theta ),\cos (\varphi )]$ the unit vector in the direction of the azimuthal angle $\theta$ and polar angle $\varphi$, and $\rho (\mathbf {x},\theta ,\varphi ,t)$ the concentration of the bundles at location $\mathbf {x}$ and time *t* with angle $\theta$ and angle $\varphi$.

Due to the symmetry of the above tensor, the tensor is orthogonal diagonalizable. Hence, the tensor can be decomposed as a sum of weighed outer products of orthonormal eigenvectors:

11 $$\begin{aligned} \Omega ^{\rho }(\mathbf {x},t) = \sum _{i = 1}^{3}\lambda _{i}(\mathbf {x},t)\left[ \mathbf {q}_{i}(\mathbf {x},t)\left( \mathbf {q}_{i}(\mathbf {x},t)\right) ^{\text {T}}\right] , \end{aligned}$$

where $(\lambda _{i},\mathbf {q}_{i})$ are the eigenpairs of the tensor. These eigenpairs can be used to measure the degree of anisotropy of the dermal layer. That is, the larger the difference between the different (positive, real) eigenvalues, the more anisotropic the dermal layer. If all eigenvalues are equal, then the dermal layer is perfectly isotropic (See also Sect. 2.4). The eigenvector corresponding to the largest eigenvalue provides the dominant direction of the collagen bundles. The concentration of the collagen molecules at location $\mathbf {x}$ and time *t* can be recovered from the above tensor by either adding its eigenvalues or determining its trace. In the remainder of the text, we order the eigenvalues such that $\lambda _{3} \ge \lambda _{2} \ge \lambda _{1}$.

We assume that the secreted collagen molecules are attached to the ECM instantaneously. Hence, the flux associated with the $(j,k){\text {th}}$ entry of the tensor $\Omega ^{\rho }$ is

12 $$\begin{aligned} \mathbf {J}_{\Omega ^{\rho }_{jk}} = \mathbf {0}. \end{aligned}$$

Furthermore, we incorporate into the model the production of collagen molecules by both fibroblasts and myofibroblasts. Similar to the mechanism proposed by Olsen et al. (1998b), we assume that a portion of the collagen molecules are deposited and reoriented in the direction of movement of the (myo)fibroblasts. The remainder of the newly secreted collagen molecules are deposited by ratio in the direction of the present collagen bundles. The ratio of the amount of molecules that are deposited in the direction of movement of the cells to the amount of molecules that are deposited in the direction of the present collagen bundles is determined by the walking speed of the cells (i.e., the magnitude of the cell fluxes). We assume that the secretion rate of the molecules is enhanced in the presence of the signaling molecule. Finally, we use the same general functional form for the removal of the collagen molecules as was used previously (Koppenol et al. 2016). This removal takes place by ratio. Taken together, we obtain for the (*j*, *k*)th entry of the tensor $\Omega ^{\rho }$

13 $$\begin{aligned}&R_{\Omega ^{\rho }_{jk}} = k_{\rho }\Bigg \{1 + \left[ \frac{k_{\rho }^{\max }c}{a_{c}^{IV} + c}\right] \Bigg \} \nonumber \\&\quad \left\{ \left[ N\left[ e^{-\beta _{\rho }\left\| \mathbf {J}_{N}\right\| }\right] + \eta M\left[ e^{-\beta _{\rho }\left\| \mathbf {J}_{M}\right\| }\right] \right] \left[ \frac{\Omega ^{\rho }_{jk}}{\text {tr}\left( \Omega ^{\rho }\right) }\right] \right. \nonumber \\&\quad \qquad +\left. N\left[ \frac{1 - e^{-\beta _{\rho }\left\| \mathbf {J}_{N}\right\| }}{\left[ \max \left( \left\| \mathbf {J}_{N}\right\| ,\gamma \right) \right] ^{2}}\right] \left[ \mathbf {J}_{N}\left( \mathbf {J}_{N}\right) ^{\text {T}}\right] _{jk} \right. \nonumber \\&\quad \qquad + \left. \eta M\left[ \frac{1 - e^{-\beta _{\rho }\left\| \mathbf {J}_{M}\right\| }}{\left[ \max \left( \left\| \mathbf {J}_{M}\right\| ,\gamma \right) \right] ^{2}}\right] \left[ \mathbf {J}_{M}\left( \mathbf {J}_{M}\right) ^{\text {T}}\right] _{jk}\right\} \nonumber \\&\quad \qquad -\,\delta _{\rho }\Bigg [\frac{\text {tr}\left( \Omega ^{\rho }\right) F}{1 + a_{c}^{III}c}\Bigg ]\Omega ^{\rho }_{jk}, \end{aligned}$$

where $k_{\rho }$ is the collagen molecule secretion rate, $k_{\rho }^{\max }$ is the maximum factor with which this rate can be enhanced due to the presence of the signaling molecule, $a_{c_{N}}^{IV}$ is the concentration of the signaling molecule that causes the half-maximum enhancement of the secretion rate, $\beta _{\rho }$ represents the sensitivity of (myo)fibroblasts to deposit and reorient (newly secreted) collagen molecules in the direction of cell movement, $\eta$ is the ratio of myofibroblasts to fibroblasts in the maximum net secretion rate of collagen molecules, and $\delta _{\rho }$ is the degradation rate of the collagen molecules. The constant $\gamma$ is a small positive value that is added to the model to prevent the division by zero in the case of no flux of either cell type. In this study, we take $\gamma = 10^{-8}\ \text {cells}/(\text {cm}^{2}\ \text {day})$.

### The force balance

Given that we model the dermal layer as a orthotropic material, we use the following general constitutive stress–strain relationship (i.e., $\mathbf {\sigma }' = \mathbf {C}'{\mathbf {\epsilon }}'$):

14 $$\begin{aligned}&\mathbf {C}' = \nonumber \\&\quad \begin{bmatrix} \frac{\left[ -1 + \nu _{23}\nu _{32}\right] E_{1}}{\Delta }&\frac{-\left[ \nu _{21} + \nu _{23}\nu _{31}\right] E_{1}}{\Delta }&\frac{-\left[ \nu _{31} + \nu _{21}\nu _{32}\right] E_{1}}{\Delta }&0&0&0 \\ \frac{-\left[ \nu _{12} + \nu _{13}\nu _{32}\right] E_{2}}{\Delta }&\frac{\left[ -1 + \nu _{13}\nu _{31}\right] E_{2}}{\Delta }&\frac{-\left[ \nu _{32} + \nu _{12}\nu _{31}\right] E_{2}}{\Delta }&0&0&0 \\ \frac{-\left[ \nu _{13} + \nu _{23}\nu _{12}\right] E_{3}}{\Delta }&\frac{-\left[ \nu _{23} + \nu _{13}\nu _{21}\right] E_{3}}{\Delta }&\frac{\left[ -1 + \nu _{12}\nu _{21}\right] E_{3}}{\Delta }&0&0&0 \\ 0&0&0&G_{23}&0&0 \\ 0&0&0&0&G_{13}&0 \\ 0&0&0&0&0&G_{12} \end{bmatrix}, \nonumber \\ \end{aligned}$$

with

15 $$\begin{aligned} \Delta= & {} \nu _{13}\nu _{21}\nu _{32} + \nu _{23}\nu _{12}\nu _{31} + \nu _{13}\nu _{31} \nonumber \\&+\,\nu _{12}\nu _{21} + \nu _{23}\nu _{32} - 1 \end{aligned}$$

and

16 $$\begin{aligned} G_{jk} = \frac{E_{j}}{2\left[ 1 + \nu _{jk}\right] }, \end{aligned}$$

where $(\mathbf {\sigma '})^{\text {T}} = [\sigma _{11},\sigma _{22},\sigma _{33},\sigma _{23},\sigma _{13},\sigma _{12}]$, $\nu _{\cdot \cdot }$ are the Poisson ratios, $E_{\cdot }$ are the Young’s moduli, $G_{\cdot \cdot }$ are the shear moduli, $(\mathbf {\epsilon '})^{\text {T}} = [\epsilon _{11},\epsilon _{22},\epsilon _{33},2\epsilon _{23},2\epsilon _{13},2\epsilon _{12}]$ (Lempriere 1968). Here, the axes of the material coordinate system coincide locally with the principal axes of the sample (i.e., the axes of the material coordinate system run parallel to the eigenvectors of the tensor $\Omega ^{\rho }$). In order to make the bulk material properties dependent on the local geometrical arrangement of the collagen bundles, we propose the following definitions for these properties:

17 $$\begin{aligned}&E_{j}(\mathbf {x},t) = E\lambda _{j}(\mathbf {x},t), \end{aligned}$$

18 $$\begin{aligned}&\nu _{jk}(\mathbf {x},t) = \nu \left[ \frac{\lambda _{j}(\mathbf {x},t)}{\sum _{l}\lambda _{l}(\mathbf {x},t)}\right] , \end{aligned}$$

where *E* and $\nu$ are constants. Using these definitions have two nice consequences: The symmetry of the elasticity tensor $\mathbf {C}'$ is guaranteed and the positivity of the stored strain energy density in the dermal layer is guaranteed. The elasticity tensor is symmetric when the equality

19 $$\begin{aligned} \frac{\nu _{jk}}{E_{j}} = \frac{\nu _{kj}}{E_{k}} \end{aligned}$$

holds for $j,k \in \{1,2,3\}$ and $j \ne k$ (Lempriere 1968). The stored strain energy density is positive when the elasticity tensor is positive definite. This is the case when the inequalities

20 $$\begin{aligned} E_{j}> 0,\quad G_{jk}> 0,\quad \Delta < 0,\quad \text {and}\quad \frac{E_{j}}{E_{k}} > \nu _{jk}^{2} \end{aligned}$$

hold (Lempriere 1968). Together, the dynamics and the initial conditions of the modeled constituents of the dermal layer imply $\lambda _{i}(\mathbf {x},t) > 0$ for all $\mathbf {x} \in \Omega _{\mathbf {x}}$, for all time *t* and $i \in \{1,2,3\}$ (with $\Omega _{\mathbf {x}}$ the domain of computation in Eulerian coordinates). Combined with the proposed definitions for the bulk material properties of the dermal layer and the values chosen for the constants *E* and $\nu$, these are sufficient conditions to guarantee that the above equalities and inequalities hold; checking this is straightforward. Hence, the elasticity tensor is indeed symmetric, positive definite, and consequently, the stored strain energy density in the system is always positive.

Notice furthermore that if the distribution of the collagen bundles is uniform (i.e., $\lambda _{1} = \lambda _{2} = \lambda _{3}$), then all Poisson ratios are equal. Likewise, all Young’s moduli and shear moduli are equal. This implies that the elasticity tensor becomes equal to the elasticity tensor of an isotropic material (Lai et al. 1999). This is a nice property because this is exactly what you would expect given the uniformity of the distribution of the collagen bundles in the dermal layer.

The tensors from Eq. (14) need to be transformed so that they coincide with the global coordinate system (i.e., the Eulerian coordinate system). Due to the made assumptions with respect to the derivatives of the cell densities (See Sect. 2.5), the chosen initial conditions for the distribution of the collagen bundles [i.e., Eq. (33)] and the included dynamics for the production of collagen molecules [i.e., Eq. (13)], the first axis of the local material coordinate system always runs parallel to the first axis of the global coordinate system. This implies that the following transformations can be used to transform the tensors from Eq. (14):

21 $$\begin{aligned} \mathbf {\sigma }'&= \mathbf {T}_{1}\mathbf {\sigma } \end{aligned}$$

22 $$\begin{aligned} {\mathbf {\epsilon }}'&= \mathbf {T}_{2}{\mathbf {\epsilon }} \end{aligned}$$

23 $$\begin{aligned} \mathbf {T}_{1}\mathbf {C}&= \mathbf {C}'\mathbf {T}_{2}, \end{aligned}$$

with

24 $$\begin{aligned} \mathbf {T}_{1} = \begin{bmatrix} 1&\quad 0&\quad 0&\quad 0&\quad 0&\quad 0 \\ 0&\quad m^{2}&\quad n^{2}&\quad -2nm&\quad 0&\quad 0 \\ 0&\quad n^{2}&\quad m^{2}&\quad 2nm&\quad 0&\quad 0 \\ 0&\quad nm&\quad -nm&\quad m^{2} - n^{2}&\quad 0&\quad 0 \\ 0&\quad 0&\quad 0&\quad 0&\quad m&\quad n \\ 0&\quad 0&\quad 0&\quad 0&\quad -n&\quad m \end{bmatrix}, \end{aligned}$$

and

25 $$\begin{aligned} \mathbf {T}_{2} = \begin{bmatrix} 1&\quad 0&\quad 0&\quad 0&\quad 0&\quad 0 \\ 0&\quad m^{2}&\quad n^{2}&\quad -nm&\quad 0&\quad 0 \\ 0&\quad n^{2}&\quad m^{2}&\quad nm&\quad 0&\quad 0 \\ 0&\quad 2nm&\quad -2nm&\quad m^{2} - n^{2}&\quad 0&\quad 0 \\ 0&\quad 0&\quad 0&\quad 0&\quad m&\quad n \\ 0&\quad 0&\quad 0&\quad 0&\quad -n&\quad m \end{bmatrix}, \end{aligned}$$

where $\mathbf {\sigma } = \mathbf {C}{\mathbf {\epsilon }}$, $(\mathbf {\sigma })^{\text {T}} = [\sigma _{xx},\sigma _{yy},\sigma _{zz},\sigma _{yz},\sigma _{xz},\sigma _{xy}]$, $({\mathbf {\epsilon }})^{\text {T}} = [\epsilon _{xx},\epsilon _{yy},\epsilon _{zz},2\epsilon _{yz},2\epsilon _{xz},2\epsilon _{xy}]$, $m = \cos (\varphi _{r})$, $n = \sin (\varphi _{r})$, and $\varphi _{r}$ is the angle of the clockwise rotation that, respectively, aligns the second and the third axis of the local material coordinate system (i.e., the second and third eigenvector of the tensor $\Omega ^{\rho }$) with the second and the third axis of the global coordinate system. (Please note that the following holds: $(\mathbf {T}_{1}(\varphi _{r}))^{-1} = \mathbf {T}_{1}(-\varphi _{r})$. This equality simplifies the calculation of the elasticity tensor $\mathbf {C}$ from Eq. (23).)

Finally, we incorporate into the model the generation of an isotropic stress by the myofibroblasts due to their pulling on the ECM. This stress is proportional to the product of the cell density of the myofibroblasts and a simple function of the concentration of the collagen molecules (Koppenol et al. 2016; Olsen et al. 1995). No other forces are incorporated into the model. Taken together, we obtain

26 $$\begin{aligned}&\mathbf {f} = \nabla \cdot \mathbf {\psi }, \end{aligned}$$

27 $$\begin{aligned}&\mathbf {\psi } = \xi M\left[ \frac{\rho }{R_{\rho }^2 + \rho ^{2}}\right] \mathbf {I}, \end{aligned}$$

where $\mathbf {\psi }$ is the total generated stress by the myofibroblast population, $\xi$ is the generated stress per unit cell density and the inverse of the unit collagen molecule concentration, and $R_{\rho }$ is a fixed constant.

### The domain of computation

We assume that $u = \partial v/\partial x = \partial w/\partial x = 0$ holds within the modeled portion of the dermal layer, with the *yz*-plane running parallel to the surface of the skin and ${\mathbf {u}} = (u,v,w)^{\text {T}}$. Furthermore, we assume that the derivatives of the cell densities and the concentrations of the modeled constituents of the dermal layer are equal to zero in the direction perpendicular to the surface of the skin. Taken together, these assumptions imply that the calculations can be performed on an arbitrary, infinitely thin slice of dermal layer oriented parallel to the surface of the skin, and that the results from these calculations are valid for every infinitely thin slice oriented parallel to the surface of the skin. Using Lagrangian coordinates ($\mathbf {X} = (X,Y,Z)^{\text {T}}$), the domain of computation $\Omega _{\mathbf {X}}$ is described by

28 $$\begin{aligned} \Omega _{\mathbf {X}} \in \{X=0, -10 \le Y\le 10, -10 \le Z\le 10\}. \end{aligned}$$

### The initial conditions and the boundary conditions

The initial conditions give a description of the cell densities and the concentrations at the onset of the proliferative phase of the wound healing cascade. For the generation of the simulation results, we use two wound shapes: a circular shape and a square shape. For the construction of these shapes, we use the following general indicator function

29 $$\begin{aligned} I\left( r,s_{1},s_{2}\right) = {\left\{ \begin{array}{ll} 0 &{} \text {if } r < \left[ s_{1} - s_{2}\right] , \\ \frac{1}{2}\left[ 1 + \sin \left( \frac{\left[ r - s_{1}\right] \pi }{2s_{2}}\right) \right] &{} \text {if } \left| r - s_{1}\right| \le s_{2}, \\ 1 &{} \text {if } r > \left[ s_{1} + s_{2}\right] . \end{array}\right. } \end{aligned}$$

The values for the parameters $s_{1}$ and $s_{2}$ determine, respectively, the location and the steepness of the boundary of the wounded area. In order to construct the circular wounds, we use the following function

30 $$\begin{aligned} w_{c}(\mathbf {X}) = I\left( \left\| \mathbf {X}\right\| ,\sqrt{16/\pi },0.10\right) , \end{aligned}$$

In order to construct square wounds with the same surface area, we use the following function

31 $$\begin{aligned} w_{s}(\mathbf {X}_{r})= & {} 1 - \left[ 1 - I\left( Y_{r},2,0.10\right) \right] \left[ 1 - I\left( Z_{r},2,0.10\right) \right] \nonumber \\&\times I\left( Y_{r},2,0.10\right) I\left( Z_{r},2,0.10\right) , \end{aligned}$$

with $\mathbf {X}_{r} = \mathbf {R}(\theta _{r})\mathbf {X} = (X_{r},Y_{r},Z_{r})^{\text {T}}$ and $\theta _{r} = \pi /4\ \text {rad}$, where $\mathbf {R}(\theta )$ is the counterclockwise rotation matrix that rotates vectors by an angle $\theta$ about the *X*-axis. For $i\in \{c,s\}$, $w_{i} = 0$ corresponds to completely wounded dermis and $w_{i} = 1$ corresponds to unwounded dermis.

Based on these general functions for the shapes of the wound, we take the following initial conditions for the cell densities and the concentration of the signaling molecules:

32 $$\begin{aligned} N(\mathbf {X},0)&= \left[ I^{w} + \left[ 1 - I^{w}\right] w_{i}\left( \mathbf {X}\right) \right] \overline{N}, \nonumber \\ M(\mathbf {X},0)&= \overline{M}, \nonumber \\ c(\mathbf {X},0)&= \left[ 1 - w_{i}\left( \mathbf {X}\right) \right] c^{w}, \end{aligned}$$

where $\overline{N}$ and $\overline{M}$ are, respectively, the equilibrium cell density of the fibroblast population and the equilibrium cell density of the myofibroblast population of the unwounded dermis. The constant $c^{w}$ represents the maximum initial concentration of the signaling molecule in the wounded area. We assume that there are some fibroblasts and collagen bundles present in the wounded area; the parameter $I^{w}$ determines the minimum amount of fibroblasts and collagen bundles present initially in the wounded area.

**Fig. 1:**
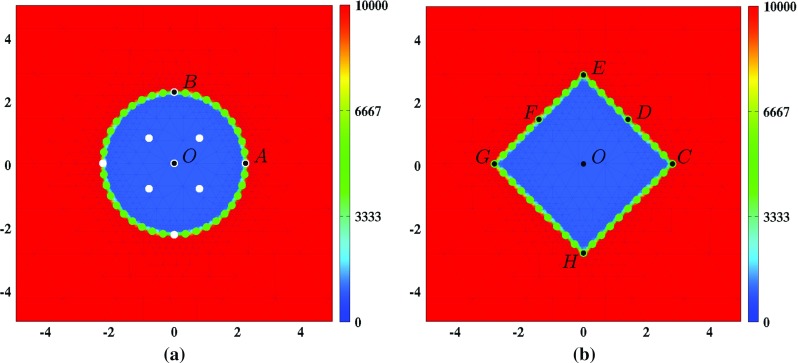
Graphical representations of the initial conditions. Depicted are in both subfigures, the initial shape of the wound and, in color scale, the initial cell density of the fibroblasts ($\text {cells}/\text {cm}^{3}$). In both subfigures, the scale along both axes is in centimeters. **a** The *white dots mark* the material points where the evolution of the cell density/concentration of the individual modeled constituents was traced over time for the generation of some of the figures in Sect. 4. In both subfigures, the *green dots mark* the material points that were used to trace the evolution over time of the surface area of the wound. That is, at each time point, the area of the polygon with vertices located at the displaced green material points has been determined. Finally, the material points labeled with a letter have been used to study the evolution over time of the shape of the wound. See Sect. 4 for more details on this matter

For the initial distribution of the collagen bundles, we take the following:

33 $$\begin{aligned}&\Omega ^{\rho }(\mathbf {X},0) = \left\{ \left[ I^{w} + \left[ 1 - I^{w}\right] w_{i}\left( \mathbf {X}\right) \right] \left\{ \frac{1}{1 + r_{a}}\left[ \hat{{\mathbf {e}}}_{X}\left( \hat{{\mathbf {e}}}_{X}\right) ^{\text {T}}\right] \right. \right. \nonumber \\&\quad \left. \left. + \frac{r_{a}}{1 + r_{a}}\left[ \frac{r_{b}}{1 + r_{b}}\left[ \hat{{\mathbf {e}}}_{Y}\left( \hat{{\mathbf {e}}}_{Y}\right) ^{\text {T}}\right] + \frac{1}{1 + r_{b}}\left[ \hat{{\mathbf {e}}}_{Z}\left( \hat{{\mathbf {e}}}_{Z}\right) ^{\text {T}}\right] \right] \right\} \right\} \overline{\rho },\nonumber \\ \end{aligned}$$

with

34 $$\begin{aligned} \hat{{\mathbf {e}}}_{j} = \mathbf {R}(\theta _{b}){\mathbf {e}}_{j}, \end{aligned}$$

for $j \in \{X,Y,Z\}$, where ${\mathbf {e}}_{j}$ is the unit vector that runs parallel to the $j{\text {th}}$ coordinate axis. The value for the parameter $r_{a}$ determines which proportion of the collagen bundles are oriented in the direction perpendicular to the surface of the skin and which proportion of the collagen bundles are running parallel to the surface of the skin. In uninjured skin, the majority of the collagen bundles of the dermal layer run parallel to the surface of the skin, while only a small portion of the fibers are oriented out-of-plane (Holzapfel 2001; Annaidh et al. 2012). Therefore, we set $r_{a}$ to a relatively large value. The values for the parameter $r_{b}$ and the angle $\theta _{b}$ together determine the overall distribution of the collagen bundles that run parallel to the surface of the skin. Over simulations, we vary the values for these latter two parameters in order to investigate the effect of such a variation on the contraction of wounds. The used (ranges of) values are depicted in Table 1 in the appendix. The parameter $\overline{\rho }$ is the equilibrium concentration of the collagen molecules in the unwounded dermis.

With respect to the initial conditions for the displacement of the dermal layer, we observe the following. The initial cell density of the myofibroblasts is equal to zero everywhere in the domain of computation. Looking at Eq. (27), this implies $\mathbf {f}(\mathbf {x},0) = \mathbf {0}$. Therefore,

35 $$\begin{aligned} {\mathbf {u}}(\mathbf {x},0) = \mathbf {0}\quad \forall \mathbf {x}\in \Omega _{x}. \end{aligned}$$

See Fig. 1 for graphical representations of the initial conditions that have been used in this study.

With respect to the boundary conditions for the modeled constituents of the dermal layer, we take the following Dirichlet boundary conditions

36 $$\begin{aligned} N = \overline{N},\ \ M = \overline{M},\ \ c = \overline{c}, \end{aligned}$$

where $\overline{c}$ is the equilibrium concentration of the signaling molecules in the unwounded dermis. Finally, we take the following Dirichlet boundary condition for the mechanical component of the model

37 $$\begin{aligned} {\mathbf {u}} = \mathbf {0}. \end{aligned}$$

### The (ranges of the) values for the parameters

Table 1 in the appendix provides an overview of the dimensional (ranges of the) values for the parameters of the model. Most of these values were either obtained directly from previously conducted studies or estimated from results of previously conducted studies. In addition, we were able to determine the values for three more parameters due to the fact that these values are a necessary consequence of the values chosen for other parameters (Koppenol et al. 2016).

**Fig. 2:**
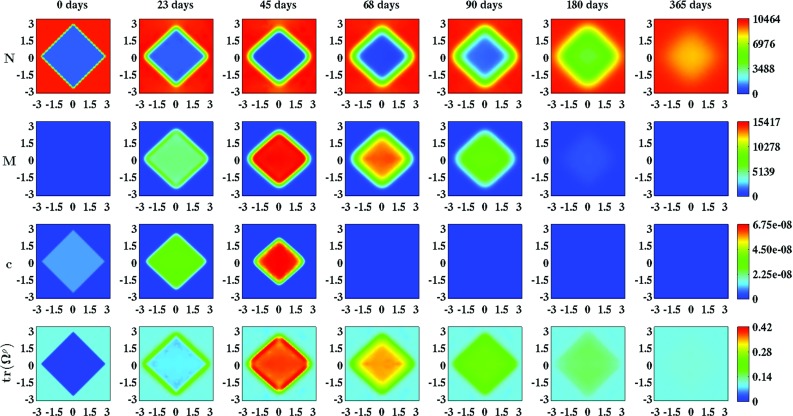
An overview of a simulation with a square wound, $r_{b} = 5$ and $\theta _{b} = 0\ \text {rad}$. The values for the remaining parameters are equal to those depicted in Table 1. The *first two rows* show the evolution over time of the cell density of, respectively, the fibroblast population and the myofibroblast population. The *color scales* represent the cell densities, measured in $\text {cells}/\text {cm}^{3}$. The *last two rows* show the evolution over time of the concentration of, respectively, the signaling molecules and the collagen molecules. The *color scales* represent the concentrations, measured in $\text {g}/\text {cm}^{3}$. Within the subfigures, the scale along both axes is in centimeters

**Fig. 3:**
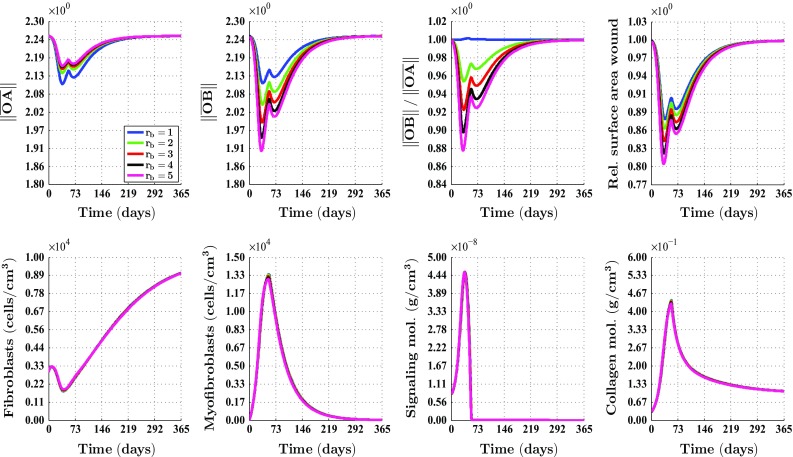
An overview of the healing response in the case of a circular wound, $\theta _{b} = 0\ \text {rad}$ and different values for the ratio $r_{b}$. The values for the remaining parameters are equal to those depicted in Table 1 in the appendix. See Fig.  1a for a depiction of the line segments $\overline{OA}$ and $\overline{OB}$, and a depiction of where the cell densities and the concentrations of the modeled constituents were measured over time. The subfigures on the *bottom row* show the averages of these latter measurements. See also Fig. 1a for a depiction of how the surface area of the wound was measured over time. The legend displayed in the top-left subfigure applies to all subfigures. Due to the fact that the curves depicted in the subfigures on the *bottom row* are situated more or less on top of each other, most of them are hardly visible

## The applied numerical algorithm

In order to express the kernel of the algorithm and generate simulation results, we used MATLAB together with MATLAB’s Parallel Computing Toolbox (The MathWorks Inc 2014). Furthermore, we interfaced the kernel consecutively with a slightly adapted version of the mesh generator developed by Persson and Strang (2004) for the generation of a base triangulation of the domain of computation, the element resolution refinement/recoarsement tool of the computational fluid dynamics (CFD) software package FEATFLOW2 for the adjustment of the resolution of the elements of the base triangulation (Turek 1998), and the scaling and permutation routine HSL_MC64 for the scaling and permutation of the linear systems (HSL 2013). The equations of the model were non-dimensionalized furthermore by applying the following non-dimensionalization:

38 $$\begin{aligned} x&= Lx^{*},&t&= \left[ L^{2}/\left[ D_{F}\overline{N}\right] \right] t^{*},&\Omega ^{\rho }&= \overline{\rho }{\Omega ^{\rho }}^{*},\nonumber \\ N&= \overline{N}N^{*},&M&= \overline{N}M^{*},&c&= c^{w}c^{*}, \nonumber \\ {\mathbf {u}}&= L{\mathbf {u}}^{*},&{\mathbf {v}}&= \left[ \left[ D_{F}\overline{N}\right] /L\right] {\mathbf {v}}^{*},&\mathbf {\sigma }&= \left[ \left[ \xi \overline{N}\right] /\overline{\rho }\right] \mathbf {\sigma }^{*}, \end{aligned}$$

where $L = 1\ \text {cm}$ is the length scale of the model. The variables with the asterisks are the non-dimensionalized variables.

In order to find a solution for the system of equations from Eq. (2), we used the method of lines together with the standard fixed-point defect correction method (Van Kan et al. 2014). The two equations of the system were solved in a segregated way. That is, each time-step approximations of the solutions for the modeled constituents of the dermal layer were determined first, and subsequently, an approximation of the solution for the displacement of the dermal layer was determined. This scheme was iterated until certain standard convergence criteria were met. For the discretization of the system of equations, the first-order backward Euler time-integration method was used together with a moving-grid finite-element method (Madzvamuse et al. 2003). On the discretized system of equations that describe the dynamics of the modeled constituents of the dermal layer, we applied a semi-implicit flux-corrected transport (FCT) limiter and a source term splitting procedure in order to enforce positivity of the approximations of the solutions for the constituents of the dermal layer (Möller et al. 2008; Patankar 1980). Finally, we used an automatically adaptive time-stepping method to select the sizes of the individual time steps (Kavetski et al. 2002).

More details about certain aspects of the applied numerical algorithm can be found in one of our previous studies (Koppenol et al. 2016). More details about the procedure surrounding the adjustment of the resolution of the elements of the base triangulation can be found in the studies by Möller (2008); Möller and Kuzmin (2006).

## Simulation results

In order to obtain some insight into the dynamics of the model, we present an overview of a simulation in Fig. 2. Figure 3 shows what the impact is of changing the initial distribution of the collagen bundles that run parallel to the surface of the skin, on the contraction of a circular wound. Due to the symmetry properties of a circular wound, it is not interesting to vary the value for the angle $\theta _{b}$. (In the case of a perfectly circular wound, changing the value for the angle $\theta _{b}$ will merely rotate the solution counterclockwisely through an angle $\theta _{b}$.) Hence, solely the value for the ratio $r_{b}$ is changed over simulations. Note that changing the value for the ratio $r_{b}$ not only influences the degree of overall contraction of the wounded area (as measured by the evolution over time of the relative surface area of the wound compared to the surface area of the wound at day 0), but also the evolution over time of the shape of the wounded area (as measured by the evolution over time of the (ratio of the) lengths of the line segments $\overline{OA}$ and $\overline{OB}$). Furthermore, note that the evolution over time of the cell densities and the concentrations of the different modeled constituents are hardly influenced by changing the value for the ratio $r_{b}$.

**Fig. 4:**
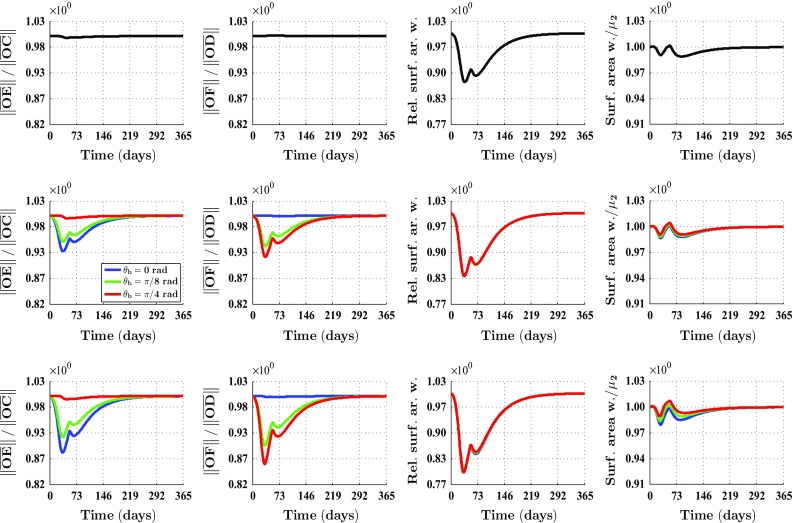
An overview of the healing response in the case of a square wound and different values for both the ratio $r_{b}$ and the angle $\theta _{b}$. The values for the remaining parameters are equal to those depicted in Table 1 in the appendix. See Fig. 1b for a depiction of the line segments $\overline{OC}$, $\overline{OD}$, $\overline{OE}$ and $\overline{OF}$. See also Fig. 1b for a depiction of how the surface area of the wound was measured over time. $\mu _{2}$ equals the surface area of the quadrilateral *CEGH*. See again Fig. 1b for a depiction of the vertices of this quadrilateral. The subfigures on the *top row* show overviews for $r_{b} = 1$ and $\theta _{b} = 0\ \text {rad}$, the subfigures on the *middle row* show overviews for $r_{b} = 3$, and the subfigures on the *bottom row* show overviews for $r_{b} = 5$. The legend displayed in the *middle row* applies to all subfigures on the *middle row* and the *bottom row*. Due to the fact that the curves depicted in the subfigures related to the relative surface area of the wound are situated more or less on top of each other, solely one curve is mostly visible

Figure 4 shows what the impact is of changing the initial distribution of the collagen bundles that run parallel to the surface of the skin, on the contraction of a square wound. (Note that if $r_{b} = 1$, then the solution is not dependent on the value for the angle $\theta _{b}$. Looking at Eq. (33), this becomes clear straight away.) Contrary to the case where the wound is circular, it is interesting to change both the value for the angle $\theta _{b}$ and the value for the ratio $r_{b}$ in the case of a square wound. Due to the symmetry properties of a square wound, it is sufficient to vary the angle $\theta _{b}$ between $0\ \text {rad}$ and $\pi /4\ \text {rad}$. (A solution for an angle larger than $\pi /4\ \text {rad}$ can be obtained from a solution for an angle between $0\ \text {rad}$ and $\pi /4\ \text {rad}$ by applying a proper reflection and/or rotation on this latter solution.) Similar to the case of a circular wound, changing the value for the ratio $r_{b}$ influences both the degree of overall contraction of the wounded area (as measured by the evolution over time of the relative surface area of the wound compared to the surface area at day 0) and the evolution over time of the shape of the wounded area (as measured by the evolution over time of the ratio of the lengths of the line segments $\overline{OC}$ and $\overline{OE}$, the ratio of the lengths of the line segments $\overline{OD}$ and $\overline{OF}$, and the surface area of the wound relative to the surface area of the quadrilateral *CEGH*).

Furthermore, Fig. 4 shows that the evolution over time of the shape of the wounded area is also influenced by the orientation of the collagen bundles relative to the position of the wound in the case of a square wound. Interestingly, this relative orientation of the collagen bundles has hardly any influence on the evolution over time of the surface area of the wound relative to the surface area at day 0. Although not depicted in Fig. 4, we want to mention here that similar to the case of a circular wound, the evolution over time of the cell densities and the concentrations of the different modeled constituents is hardly influenced by changing the values for the ratio $r_{b}$ and the angle $\theta _{b}$.

**Fig. 5:**
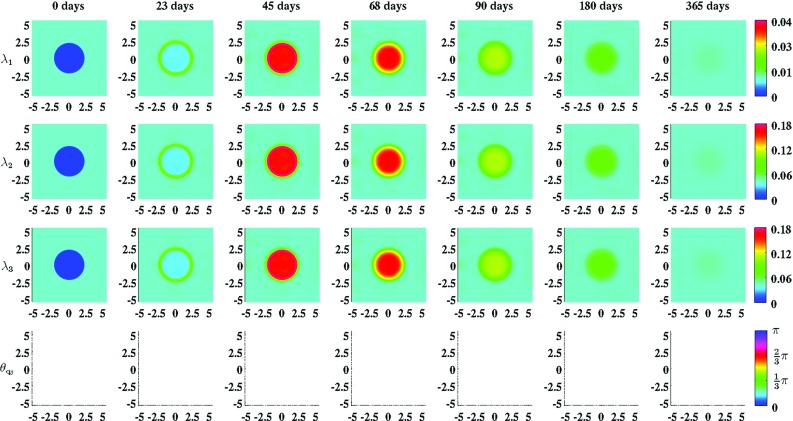
An overview of the evolution over time of the geometrical arrangement of the collagen bundles during the healing of a circular wound. For the generation of these simulation results, we took $r_{b} = 1$, $\theta _{b} = 0\ \text {rad}$ and $\beta _{\rho } = 0\ (\text {cm}^{2}\ \text {day})/\text {cells}$. The values for the remaining parameters are equal to those depicted in Table 1. $\lambda _{1}$, $\lambda _{2}$, and $\lambda _{3}$ are the eigenvalues of the tensor $\Omega ^{\rho }$ and $\theta _{\mathbf {q}_{3}}$ is the angle between the eigenvector related to the largest eigenvalue $\lambda _{3}$ (where the third element of this eigenvector is larger than/equal to zero) and the positive horizontal axis. Within the subfigures on the *bottom row*, the transparency (i.e., $\alpha (\mathbf {x},t)$) is set to either zero (opaque) or one (fully transparent) based on the following rule: if $(\lambda _{3}(\mathbf {x},t) - \lambda _{2}(\mathbf {x},t))/\lambda _{2}(\mathbf {x},t) > 0.2$, then $\alpha (\mathbf {x},t) = 0$, else $\alpha (\mathbf {x},t) = 1$

**Fig. 6:**
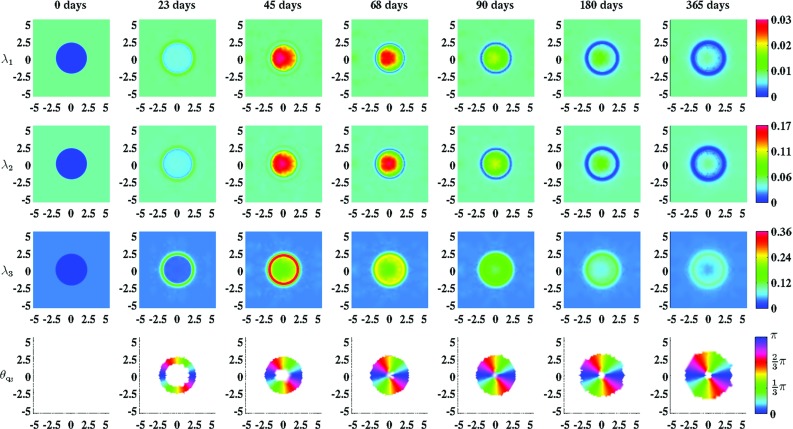
An overview of the evolution over time of the geometrical arrangement of the collagen bundles during the healing of a circular wound. For the generation of these simulation results, we took $r_{b} = 1$, $\theta _{b} = 0\ \text {rad}$ and $\beta _{\rho } = 10^{-1}\ (\text {cm}^{2}\ \text {day})/\text {cells}$. The values for the remaining parameters are equal to those depicted in Table 1. $\lambda _{1}$, $\lambda _{2}$, and $\lambda _{3}$ are the eigenvalues of the tensor $\Omega ^{\rho }$ and $\theta _{\mathbf {q}_{3}}$ is the angle between the eigenvector related to the largest eigenvalue $\lambda _{3}$ (where the third element of this eigenvector is larger than/equal to zero) and the positive horizontal axis. Within the subfigures on the *bottom row* the transparency (i.e., $\alpha (\mathbf {x},t)$) is set to either zero (opaque) or one (fully transparent) based on the following rule: if $(\lambda _{3}(\mathbf {x},t) - \lambda _{2}(\mathbf {x},t))/\lambda _{2}(\mathbf {x},t) > 0.2$, then $\alpha (\mathbf {x},t) = 0$, else $\alpha (\mathbf {x},t) = 1$

Finally, Figs. 5 and 6 show the evolution over time of the geometrical arrangement of the collagen bundles during the healing of a circular wound. In order to demonstrate the effect of either including the deposition/reorientation of collagen molecules in the direction of cell movement or not, we set $\beta _{\rho }$ to zero for the generation of the simulation results in Fig. 5 and set $\beta _{\rho }$ to the standard value depicted in Table 1 for the generation of the simulation results in Fig. 6. (See the text next to Eq. (13) for a description of the parameter $\beta _{\rho }$.) If $\beta _{\rho } = 0\ (\text {cm}^{2}\ \text {day})/\text {cells}$, then this will result ultimately in newly generated tissue with a collagen bundle distribution that is exactly equal to the collagen bundle distribution of the surrounding uninjured tissue. If $\beta _{\rho } = 10^{-1}\ (\text {cm}^{2}\ \text {day})/\text {cells}$, then ultimately the majority of the collagen molecules ends up permanently oriented toward the center of the wound and in the plane that runs parallel to the surface of the skin.

## Discussion

We have presented a continuum hypothesis-based model for the simulation of the collagen bundle distribution-dependent contraction and retraction of healing dermal wounds that cover a large surface area. In order to simulate the contraction and subsequent retraction of these wounds, some of the subprocesses that take place during the proliferative and remodeling phase of the wound healing cascade were incorporated into the model. Since wound contraction mainly takes place in the dermal layer of the skin, solely a portion of this layer is included explicitly into the model. This portion of dermal layer is modeled as a heterogeneous, orthotropic continuous solid with bulk mechanical properties that are locally dependent on both the local concentration and the local geometrical arrangement of the collagen bundles.

The following four constituents of the dermal layer were selected furthermore as primary model variables: fibroblasts, myofibroblasts, generic signaling molecules, and collagen bundles. The functional forms for the movement of the cells, the biochemical kinetics associated with these cells, the dispersion of the generic signaling molecule and the release, consumption, and removal of the generic signaling molecule are identical to the functional forms used previously (Koppenol et al. 2016).

With respect to the way that the cell differentiation of fibroblasts into myofibroblasts has been incorporated into the presented model, we want to place some remarks here. Similar to Olsen et al. (1995), we assume that the rate of cell differentiation is dependent on the concentration of the signaling molecule with no differentiation taking place in the absence of the signaling molecule. Like others such as Murphy et al. (2012) and Valero et al. (2014), we are aware of the fact that this cell differentiation can only take place under conditions of sufficient mechanical stiffness. However, as is also clearly pointed out by Van de Water et al. (2013), it is unclear at present what the actual stiffness is that is perceived by fibroblasts. Furthermore, recent experimental studies have shown that the differentiation of fibroblasts into myofibroblasts is also critically dependent on the presence of particular isoforms of fibronectin (Van de Water et al. 2013). Taken together, these issues imply that the incorporation of the cell differentiation mechanism into a mathematical model in a realistic way is basically impossible at present. Hence, while being aware of the fact that the cell differentiation process is a very complex process in reality, we decided to keep things relatively simple in this modeling study.

For the representation of the collagen bundles, we used a tensorial approach similar to the one proposed by Barocas and Tranquillo (1997) and Cumming et al. (2010). Compared to the approaches developed by Dallon et al. (1999, (2001, (2000); McDougall et al. (2006) and Olsen et al. (1999, (1998b), this approach has some advantages. Dallon et al. use a vector-based representation for the representation of collagen bundles in their models. Using this vector-based representation has two major disadvantages (Cumming et al. 2010). Firstly, it does not provide any information about the degree of isotropy of the collagen bundles at individual material points within the dermal layer. Secondly, due to the use of a vector representation, collagen bundles are treated basically as unidirectional entities. Given that fiber alignment is bidirectional, this may lead to duality when one wishes to determine the degree of alignment of collagen bundles. These two disadvantages are overcome by using a tensorial approach. Furthermore, Olsen et al. model the alignment of collagen bundles in their models by considering two orthogonal configurations and assuming that the transition between these two configurations is a dynamic and reversible process. The major disadvantage of this latter approach is that this representation cannot provide an accurate representation of the orientation of the collagen bundles when this orientation is continuously distributed. The tensorial approach does not suffer from this drawback.

With respect to the dynamic change of the geometrical arrangement of the collagen bundles it is assumed that a portion of the collagen molecules are deposited and reoriented in the direction of movement of the (myo)fibroblasts (Olsen et al. 1998b). The remainder of the newly secreted collagen molecules are deposited by ratio in the direction of the present collagen bundles. The ratio of the amount of molecules that are deposited in the direction of movement of the cells to the amount of molecules that are deposited in the direction of the present collagen bundles is determined by the walking speed of the cells.

With the developed model, it is possible to simulate some general qualitative features of the dermal wound healing response (Baum and Arpey 2005; Enoch and Leaper 2007; Li et al. 2007; Monaco and Lawrence 2003). The restoration of the presence of fibroblasts in the wounded area can be simulated. Furthermore, the initial expansion and subsequent reduction of the myofibroblast population in the wounded area during the execution of the wound healing response can be simulated, and it is possible to simulate the restoration of a collagen-rich ECM in the recovering wounded area. In accordance with experimental observations (Zuijlen et al. 2003; Welch et al. 1990), it is also possible with this model to simulate the permanent increase in the proportion of the collagen bundles that runs parallel to the surface of the skin as a consequence of the execution of the wound healing process. Finally, the contraction and the subsequent retraction of the wounded area can also be simulated.

With respect to the simulation of the collagen bundle distribution-dependent contraction and subsequent retraction of healing dermal wounds, the following can be observed. Figures 3 and 4 show clearly the impact of changing the initial distribution of the collagen bundles that run parallel to the surface of the skin. The distribution of the collagen bundles influences the evolution over time of both the shape of the wounded area and the degree of overall contraction of the wounded area. Interestingly, Fig. 3 shows that these effects are solely a consequence of alterations in the initial overall distribution of the collagen bundles, and not a consequence of alterations in the evolution over time of the different cell densities and concentrations of the modeled constituents. Furthermore, it is very interesting to observe in Fig. 4 that the evolution over time of the shape of the wound is also influenced by the orientation of the collagen bundles relative to the position of the wound, while this relative orientation does not influence the evolution over time of the relative surface area of the wound compared to the surface area of the wound at day 0.

Figures 5 and 6 show clearly the effect of either including into the model the dependence of the geometrical arrangement of collagen bundles on the movement of cells or not. If it is included, then an increased portion of the collagen bundles ultimately ends up permanently in the plane running parallel to the surface of the skin, and the majority of these bundles are oriented toward the center of the wound. If the deposition/reorientation of collagen molecules in the direction of cell movement is not included, then all the newly secreted collagen molecules are deposited by ratio in the direction of the present collagen bundles. As demonstrated in Fig. 5, this will result ultimately in newly generated tissue with a collagen bundle distribution that is exactly equal to the collagen bundle distribution of the surrounding uninjured tissue.

As has been mentioned before, the fact that an increased portion of the collagen bundles ultimately ends up permanently in the plane running parallel to the surface of the skin and the fact that the majority of these bundles are oriented toward the center of the wound when the dependence of the direction of deposition/reorientation of collagen molecules on the movement of cells is included into the model, are in accordance with experimental observations (Zuijlen et al. 2003; Welch et al. 1990). This is an interesting observation. To the best of our knowledge, it is unknown at present which wound healing mechanisms cause these experimental observations. In the presented model, the dynamics related to the geometrical arrangement of the collagen bundles are dependent on the speed and the direction of movement of the (myo)fibroblasts. This direction and speed of movement are dependent subsequently on the gradient of the concentration of the signaling molecule and the evolution over time of the distribution of the cell densities. Taken together, the results obtained with the presented model suggest that the geometrical arrangement of collagen bundles in scar tissue might be altered by changing the gradient of the concentration of the signaling molecule and/or the evolution over time of the distribution of the cell densities and therewith the direction and speed of movement of the (myo)fibroblasts, during wound healing. Given that the geometrical arrangement of the collagen bundles has a huge impact on the response of dermal tissues to mechanical forces (Jor et al. 2011), this is an interesting suggestion because we think that its offers new ideas for a better treatment of deep dermal wounds that results in scar tissue that is more akin to the original tissue. We can imagine that it must be possible to investigate this suggestion further by means of an experimental study with a tissue equivalent.

Our ultimate goal is to construct mathematical models with which the structural properties of healing wounds can be predicted accurately. Since collagen bundle distribution-dependent contraction and subsequent retraction are important components of the wound healing response in the case of deep dermal wounds that cover a large surface area, we consider the development of the presented model as a step toward the construction of such predictive models.

Obviously, the presented model can be expanded in different ways. One relatively simple addition to the model would be, for instance, to make the direction of cell movement also dependent on the orientation of the collagen bundles. Previous experimental studies have demonstrated that fibroblasts align with collagen bundles, which subsequently influences the direction of movement of these cells (Guido and Tranquillo 1993). In order to incorporate this effect into the presented model, we could, for example, replace Eqs. (3) and (4) with

39 $$\begin{aligned} \mathbf {J}_{N}&= -D_{F}F\Omega ^{c}\nabla N + \chi _{F}N\nabla c, \end{aligned}$$

40 $$\begin{aligned} \mathbf {J}_{M}&= -D_{F}F\Omega ^{c}\nabla M + \chi _{F}M\nabla c, \end{aligned}$$

where

41 $$\begin{aligned} \Omega ^{c} = \frac{\Omega ^{\rho }}{\text {tr}\left( \Omega ^{\rho }\right) }. \end{aligned}$$

In addition, Verhaegen et al. (2012) have demonstrated that the stretching of both healthy skin and scar tissue induces permanent adaptation of the orientation of the collagen bundles in these tissues. Therefore, it might be interesting to incorporate into the presented model the reorientation of collagen bundles due to forces. This could be accomplished perhaps by absorbing into the presented model some of the ideas that were used for the development of the model by Olsen et al. (1999). Furthermore, it might also be interesting to add to the presented model a morphoelastic component. Adding this component to the model will make it possible to simulate the often present permanent deformation of recovering dermal tissues. The incorporation of such an effect into the current model could be accomplished, for instance, by using the morphoelastic framework developed by Hall (2009). Finally, it is probably also very interesting to investigate in a three-dimensional portion of dermal layer what would happen to the shapes of wounds and the geometrical arrangement of collagen bundles during healing when the assumptions made in Sect. 2.5 are removed.

## Appendix: The parameter value estimates

See Table 1.

**Table 1:** An overview of the dimensional (ranges of the) values for the parameters of the model

Parameter	Value	Dimensions	Reference
$D_{F}$	$$10^{-7}\ $$	$\text {cm}^{5}/(\text {cells day})$	Sillman et al. (2003)
$\chi _{F}$	$$2\times 10^{-3}\ $$	$\text {cm}^{5}/(\text {g day})$	Murphy et al. (2012)
*q*	$$-4.2\times 10^{-1}\ $$	−	NC
$r_{F}$	$$9.24\times 10^{-1}\ $$	$\text {cm}^{3q}/(\text {cells}^{q}\ \text {day})$	Ghosh et al. (2007)
$r_{F}^{\max }$	$$2\ $$	−	Strutz et al. (2001)
$a_{c}^{I}$	$$10^{-8}\ $$	$\text {g}/\text {cm}^{3}$	Grotendorst (1992)
$\kappa _{F}$	$$10^{-6}\ $$	$\text {cm}^{3}/\text {cells}$	Vande Berg et al. (1989)
$k_{F}$	$$5.4\times 10^{6}\ $$	$\text {cm}^{3}/(\text {g day})$	Desmoulière et al. (1993)
$\delta _{N}$	$$2\times 10^{-2}\ $$	$/\text {day}$	Olsen et al. (1995)
$\delta _{M}$	$$2\times 10^{-2}\ $$	$/\text {day}$	Koppenol et al. (2016)
$D_{c}$	$$2.9\times 10^{-3}\ $$	$\text {cm}^{2}/\text {day}$	Murphy et al. (2012)
$k_{c}$	$$4\times 10^{-13}\ $$	$\text {g}/(\text {cells day})$	Olsen et al. (1995)
$\eta$	$$2\ $$	−	Rudolph and Vande Berg (1991); Moulin et al. (1998)
$a_{c}^{II}$	$$10^{-8}\ $$	$\text {g}/\text {cm}^{3}$	Olsen et al. (1995)
$\delta _{c}$	$$5\times 10^{-4}\ $$	$\text {cm}^{6}/(\text {cells g day})$	Olsen et al. (1995)
$a_{c}^{III}$	$$2\times 10^{8}\ $$	$\text {cm}^{3}/\text {g}$	Overall et al. (1991)
$k_{\rho }$	$$6\times 10^{-8}\ $$	$\text {g}/(\text {cells day})$	NC
$k_{\rho }^{\max }$	$$10\ $$	−	Olsen et al. (1998a)
$a_{c}^{IV}$	$$10^{-9}\ $$	$\text {g}/\text {cm}^{3}$	Roberts et al. (1986)
$\beta _{\rho }$	$$10^{-1}\ $$	$(\text {cm}^{2}\ \text {day})/\text {cells}$	TW
$\delta _{\rho }$	$$6\times 10^{-6}\ $$	$\text {cm}^{6}/(\text {cells g day})$	Koppenol et al. (2016)
*E*	$$3.00\times 10^{2}\ $$	$(\text {N cm})/\text {g}$	Liang and Boppart (2010)
$\nu$	$$4.9\times 10^{-1}\ $$	−	Liang and Boppart (2010)
$\xi$	$$2\times 10^{-4}\ $$	$(\text {N g})/(\text {cells cm}^{2})$	Javierre et al. (2009); Wrobel et al. (2002)
$R_{\rho }$	$$3\times 10^{-1}\ $$	$\text {g}/\text {cm}^{3}$	Olsen et al. (1995)
$\overline{N}$	$$10^{4}\ $$	$\text {cells}/\text {cm}^{3}$	Olsen et al. (1995)
$\overline{M}$	$$0\ $$	$\text {cells}/\text {cm}^{3}$	Olsen et al. (1995)
$\overline{c}$	$$0\ $$	$\text {g}/\text {cm}^{3}$	NC
$\overline{\rho }$	$$10^{-1}\ $$	$\text {g}/\text {cm}^{3}$	Olsen et al. (1995)
$I^{w}$	$$10^{-1}\ $$	−	TW
$c^{w}$	$$10^{-8}\ $$	$\text {g}/\text {cm}^{3}$	Olsen et al. (1995)
$r_{a}$	$$9\ $$	−	Annaidh et al. (2012); Holzapfel (2001)
$r_{b}$	$$1-5\ $$	−	TW
$\theta _{b}$	$$0-\pi /4\ $$	$\text {rad}$	TW

The last column contains the references to the articles that were used for obtaining (estimates of) the values for the parameters. If (the range of) the value for a parameter was estimated in this study, then this is indicated by the abbreviation TW. If the value for a parameter is a necessary consequence of the values chosen for the other parameters, then this is indicated by the abbreviation NC
